# Effect of Ubiquinol on Glaucomatous Neurodegeneration and Oxidative Stress: Studies for Retinal Ganglion Cell Survival and/or Visual Function

**DOI:** 10.3390/antiox9100952

**Published:** 2020-10-03

**Authors:** Genea Edwards, Yonghoon Lee, Martha Kim, Soham Bhanvadia, Keun-Young Kim, Won-Kyu Ju

**Affiliations:** 1 Hamilton Glaucoma Center and Shiley Eye Institute, Viterbi Family Department of Ophthalmology, University of California San Diego, La Jolla, CA 92039, USA; gedwards@umkc.edu (G.E.); nleeyh@gmail.com (Y.L.); marthakim22@gmail.com (M.K.); shbhanva@ucsd.edu (S.B.); 2 Department of Ophthalmology, Dongguk University Ilsan Hospital, Ilsandong-gu, Goyang-si 10326, Korea; 3 National Center for Microscopy and Imaging Research, Department of Neurosciences, University of California San Diego, La Jolla, CA 92039, USA; keunyoung@ncmir.ucsd.edu

**Keywords:** ubiquinol, coenzyme Q10, glaucoma, oxidative stress, mitochondria, retinal ganglion cell, BAX, OXPHOS (oxidative phosphorylation), optomotor response, visual evoked potential

## Abstract

Oxidative stress is one of major causal factors in glaucomatous neurodegeneration. Ubiquinol promotes retinal ganglion cell (RGC) survival against glaucomatous insults such as oxidative stress. Here we investigated the effect of ubiquinol on RGC survival and/or visual function in mouse models of glaucoma and oxidative stress. DBA/2J and age-matched DBA/2J-*Gpnmb^+^* (D2-*Gpnmb^+^*), which do not develop intraocular pressure elevation, or C57BL/6J mice were fed with ubiquinol (1%) or control diet daily for 5 or 2 months. We assessed RGC survival by Brn3a immunohistochemistry and measured expression levels of active and total BAX, peroxisome proliferator-activated receptor-gamma coactivator 1α, transcription factor A (TFAM) and oxidative phosphorylation (OXPHOS) complex protein. Following induction of oxidative stress by paraquat injection, we also assessed visual function. In glaucomatous retina, ubiquinol supplementation significantly promoted RGC survival, blocked BAX activation and increased TFAM and OXPHOS complex II protein expression. Also, ubiquinol supplementation ameliorated oxidative stress-induced visual dysfunction. These findings indicate that ubiquinol promotes RGC survival by increasing TFAM expression and OXPHOS complex II activity in glaucomatous neurodegeneration, and that ubiquinol enhances RGC survival and preserves visual function against oxidative stress. We propose that ubiquinol has a therapeutic potential for treating oxidative stress-associated glaucomatous neurodegeneration.

## 1. Introduction

Glaucoma is a multifactorial optic neuropathy that is characterized by a progressive degeneration of retinal ganglion cell (RGC) axons, resulting in RGC death and visual dysfunction. To date, although intraocular pressure (IOP) is the only proven manageable risk factor for glaucomatous neurodegeneration, lowering IOP often is not sufficient for interrupting glaucoma progression. The risk factors contributing to RGC degeneration are currently not well understood. Oxidative stress has been considered to be critical to the pathophysiological mechanism for mitochondrial dysfunction and RGC death in glaucomatous neurodegeneration.

Coenzyme Q10 (CoQ_10_) is thought to be as a potent antioxidant and neurotherapeutic agent. Indeed, accumulating evidence supports its effectiveness to protect neuronal cells in neurodegenerative diseases including Alzheimer’s and Parkinson’s diseases. CoQ_10_, a small lipid-soluble molecule, is endogenously synthesized and located in the mitochondrial inner membrane [1,2,3]. CoQ_10_, which is an essential cofactor of the electron transport chain system, maintains the mitochondrial membrane potential, supports ATP synthesis and inhibits reactive oxygen species generation, leading to the protection of neuronal cells. Previous studies have demonstrated that CoQ_10_ ameliorated retinal cell death in vivo and in vitro against oxidative stress or elevated IOP [4,5,6]. More recently, we have demonstrated that CoQ_10_ protects RGCs against oxidative stress or glutamate excitotoxicity, and prevented alteration of mitochondria in mouse models of glaucoma and ischemic retina in vivo and in optic nerve head (ONH) astrocytes in vitro [7,8,9].

Recent evidence showed that ubiquinol, the reduced and active form of CoQ_10_, is protective in neuronal cells in several neurodegenerative diseases including Alzheimer’s disease, traumatic brain injury, and multiple system atrophy [10,11,12,13]. Importantly, emerging evidence from our group indicates that ubiquinol diet supplementation protects RGCs via modulating the BAX/BAD/BCL-xL-mediated apoptotic pathway in acute IOP elevation-induced ischemic retinal degeneration. Since we have demonstrated that dysfunctional mitochondria induced by glaucomatous insults such as elevated IOP and oxidative stress are critical to glaucomatous RGCs degeneration, these results collectively suggest the possibility that ubiquinol would have a promising therapeutic potential to enhance RGC protection against ischemic or glaucomatous neurodegeneration.

In the present study, we investigated whether ubiquinol supplementation promotes RGC survival via modulating transcription factor A (TFAM) expression and oxidative phosphorylation (OXPHOS), and blocking BAX activation in glaucomatous neurodegeneration, as well as preserves visual function against oxidative stress.

## 2. Materials and Methods

### 2.1. Animals

Adult male and female DBA/2J and age-matched control DBA/2J-*Gpnmb^+^* (D2-*Gpnmb^+^*) (The Jackson Laboratory, Bar Harbor, ME, USA), and C57BL/6J mice (The Jackson Laboratory; S12063) were housed in covered cages, fed with a standard rodent diet ad libitum, and kept on a12 h light/12 h dark cycle. All procedures were managed in accordance with the Association for Research in Vision and Ophthalmology Statement for the Use of Animals in Ophthalmic Vision Research and under animal protocols (S12063) approved by Institutional Animal Care and Use Committee at the University of California, San Diego (CA, USA).

### 2.2. Induction of Oxidative Stress

Four month-old C57BL/6J mice were anesthetized with a mixture of ketamine (100 mg/kg, Ketaset; Fort Dodge Animal Health, Fort Dodge, IA, USA) and xylazine (9 mg/kg, TranquiVed; Vedeco, Inc., St. Joseph, MO, USA) by intraperitoneal (IP) injection. For RGC counting, eyes were pretreated with 1% proparacaine drops and 5 mM paraquat (PQ; 1 μL, Sigma-Aldrich, St. Louis, MO, USA) was used to inject into the vitreous humor using a Hamilton syringe with 34-gauge needle. Intravitreal injections were performed slowly over 1 min and the needle was kept in position for an additional 10 min to minimize leaks through the injection tract. RGC counting was assessed at 1 week after PQ injection. For optomotor response test and visual evoked potential (VEP) measurement, mice were received IP injection of PQ (5 mg/kg, Sigma-Aldrich) in saline solution two times during 1 week of period to avoid ocular tissue damages. Optomotor response and VEP measurements were performed at 1 week after PQ injection.

### 2.3. Pharmacological Treatment

Ubiquinol was obtained from Kaneka Nutrients (Pasadena, TX, USA). A standard AIN-93G purified control diet or a diet supplemented with ubiquinol (1%) in the chow were formulated by Envigo (Indianapolis, IN, USA) [14]. For a chronic IOP elevation, we studied four groups of mice as follows: a group of D2-*Gpnmb^+^* mice fed with control diet (*n* = 15 mice), a group of DBA/2J mice fed with control diet (*n* = 30 mice), a group of D2-*Gpnmb^+^* mice fed with 1% Ubiquinol diet [(*v/v*), which equals a daily dose of 1600–2000 mg/kg body weight in 25–30 g mice, *n* = 15 mice] and a group of DBA/2J mice fed with 1% Ubiquinol diet (*n* = 30 mice). For oxidative stress, we studied four groups of mice as followings: a group of non-ischemic C57BL/6J mice fed with control diet (*n* = 20 mice), a group of ischemic C57BL/6J mice fed with control diet (*n* = 20 mice), a group of non-ischemic C57BL/6J mice fed with 1% Ubiquinol diet [(*v/v*), which equals a daily dose of 1600–2000 mg/kg body weight in 25–30 g mice, *n* = 20 mice] and a group of ischemic C57BL/6J mice fed with 1% Ubiquinol diet (*n* = 20 mice).

### 2.4. IOP Measurement

The onset of IOP elevation typically occurred between 5 and 7 months of age and the peak of IOP elevation was induced by 9 to 10 months of age [15,16]. IOP elevation-induced optic nerve axon degeneration is well advanced and confirmed [15,16]. Previous studies have demonstrated that axonal loss in 10 month-old glaucomatous DBA/2J mice was prominent by confirming the presence of acquired optic neuropathy [17,18]. Single IOP measurement was performed in each of the 10 month-old DBA/2J and non-glaucomatous control D2-*Gpnmb^+^* mice and we confirmed spontaneous IOP elevation exceeding 25 mmHg (*n* = 30, DBA/2J; *n* = 15, D2-*Gpnmb^+^*). Homozygous D2-*Gpnmb^+^* mice that have a functional allele of *Gpnmb* do not develop an elevated IOP. IOP was assessed with a tonometer (Icare TONOVET, Vantaa, Finland) by measuring six times and the average was calculated automatically. Three measurements were performed and averaged per eye.

### 2.5. Tissue Preparation

Mice were anesthetized with IP injection of a mixture of ketamine/xylazine, as described, and then mice were perfused transcardially with 0.9% saline followed by 4% paraformaldehyde (Sigma-Aldrich) in phosphate buffered saline (PBS, pH 7.4, Sigma-Aldrich). Following enucleation of eyeballs, the eyes were fixed with 4% paraformaldehyde in PBS for 4 h at 4 °C. The retinas were prepared as flattened whole-mounts for immunohistochemical analysis or used immediately for Western blot analysis.

### 2.6. Whole-Mount Immunohistochemistry

Flattened retinal whole-mounts were immersed in 30% sucrose in PBS for 24 h at 4 °C. The retinas were treated with blocking solution (PBS containing 3% donkey serum, 1% bovine serum albumin, 1% fish gel and 0.1% Triton X-100) for 1 h at room temperature. The retinas were incubated with goat polyclonal Brn3a antibody (1:500; sc-31984, Santa Cruz Biotechnology, Dallas, TX, USA) for 3 days at 4 °C. After washing with PBS for 10 min by 3 times, the retinas were incubated with the secondary antibodies, Alexa Fluor-568 donkey anti-goat IgG antibody (1:100; A-11057, Invitrogen, Carlsbad, CA, USA) for 24 h, and subsequently washed with PBS.

### 2.7. Quantitative Analysis for RGC Counting

The images for Brn3a-positive RGCs were taken at 20x under fluorescence microscopy using a Nikon ECLIPSE microscope (E800; Nikon Instruments Inc., Melville, NY, USA) equipped with digital camera (SPOT Imaging, Diagnostic Instruments Inc., Sterling Heights, MI, USA). The exposures were the same for all tissue sections and were acquired using Simple PCI version 6.0 imaging software (Compix Inc., Tualatin, CA, USA). To count Brn3a-positive RGCs, we used ImageJ software (http://rsb.info.nih.gov/ij/, National Institute of Health, Baltimore, MD, USA) and measured Brn3a-positive RGC densities in 8 distinct middle areas (three-sixths of retinal radius per retinal quadrant) per retina by two investigators in a masked fashion, and the scores of RGC densities were averaged [15,19].

### 2.8. Western Blot Analysis

The retinas were homogenized in a glass-Teflon Potter homogenizer in RIPA lysis buffer (150 mM NaCl, 1 mM EDTA, 1% NP-40, 0.1% SDS, 1 mM DTT, 0.5% sodium deoxycholate and 50 mM Tri-Cl, pH 7.6) containing complete protease inhibitors (Sigma-Aldrich). Each retina extract sample (10 µg; *n* = 3 retinas/experimental group) was separated by PAGE and electrotransferred to polyvinylidenedifluoride membrane. Following blocking with 5% nonfat dry milk/0.5% Tween-20/PBS (PBS-T) for 1 h, the membrane was incubated with the primary antibodies for 16 h at 4 °C. The primary antibodies include mouse monoclonal anti-total BAX antibody (1:1000; N-20: sc-493, Santa Cruz Biotechnology), rabbit polyclonal anti-active BAX antibody (1:1000; 6A7: sc-23959, Santa Cruz Biotechnology), mouse monoclonal anti-total OXPHOS complex antibody (1:4000; 458099, Life Technologies, Carlsbad, CA, USA), rabbit polyclonal anti-peroxisome proliferator-activated receptor-gamma coactivator (PGC)-1α (1:3000; sc-13067, Santa Cruz Biotechnology), rabbit polyclonal anti-transcription factor A (TFAM) antibody (1:3000; sc-23588, Santa Cruz Biotechnology), and mouse monoclonal anti-actin antibody (1:10,000; MAB1501, Millipore, Burlington, MA, USA). After washing with PBST for 10 min by 3 times, the membranes were incubated with peroxidase-conjugated goat anti-mouse IgG (1:5000; Bio-Rad Laboratories, Hercules, CA, USA) or goat anti-rabbit IgG (1:5000; Bio-Rad Laboratories) for 2 h at room temperature. The blots were developed using chemiluminescence detection (ECL Plus; GE Healthcare Bio-Science, Pittsburgh, PA, USA) and analyzed using ImageQuant LAS 4000 and Image Quant TL 8.1 Software Package (GE Healthcare Bio-Sciences). The normalization of protein band densities was done by the protein band densities for actin.

### 2.9. Virtual Optomotor Response Analysis

Virtual optomotor response was measured in a virtual optomotor system (OptoMotry; CerebralMechanics, Lethbride, Alberta, Canada) as previously described [19,20]. Mice were placed on an unrestricted platform in the center of a virtual cylinder comprised of four monitors arranged in a square (arena) that project a sinusoidal grating (i.e., white versus black vertical bars) rotating at 12 deg/s. Mice were monitored by a camera mounted at the top of the arena while a cursor placed on the forehead centers the rotation of the cylinder at the animal’s viewing position. To assess visual acuity, tracking was determined when the mouse stops moving its body and only head-tracking movement is observed. Spatial frequency threshold, which is a measure of visual acuity, was determined automatically with accompanying OptoMotry software, which uses a step-wise paradigm based upon head-tracking movements at 100% contrast. Spatial frequency began at 0.042 cyc/deg, which gradually increased until head movement was no longer observed.

### 2.10. VEP Analysis

Mice were adapted in the dark procedure room of the vivarium for less than 12 h and prepared for recording under dim red light. Following anesthetization with IP injection of a mixture of ketamine/xylazine as described above, pupils were dilated using equal parts of topical tropicamide (1%) and phenylephrine (2.5%). As a topical anesthetic, we used proparacaine (0.5%) to avoid blinking and a drop of lubricant was applied on the cornea to prevent dehydration. Following the incision of the scalp skin, a metal electrode was inserted into the primary visual cortex through the skull (0.8 mm deep from the cranial surface and 2.3 mm lateral to the lambda). As a reference and ground, a platinum subdermal needle (Grass Telefactor) was inserted through the animal’s mouth and tail, respectively. The measurements commenced when the baseline waveform became stable, 10–15 s after attaching the electrodes. Flashes of light at 2 log cd.s/m2 were delivered through a full-field Ganzfeld bowl at 2 Hz. Following the signal was amplified, digitally processed by the software (VERIS, Electro-Diagnostic Imaging, Inc., Redwood, CA, USA), and then exported, the peak-to-peak responses were analyzed in Excel (Microsoft, Redmond, WA, USA). To isolate VEP of the measured eye from the crossed signal originating in the contralateral eye, the other eye that was not undergoing measurement was covered with a black aluminum foil eyepatch. For each eye, peak-to-peak response amplitude of the major component P1-N1 in IOP eyes was compared to that of their contralateral non-IOP controls. All the recordings were performed with the same stimulus intensity. The average signals were compared with respect to both amplitudes [19,21].

### 2.11. Statistical Analysis

Data were expressed as the mean ± SEM. For multiple group comparisons, we used either one-way ANOVA or two-way ANOVA, using GraphPad Prism (GraphPad, San Diego, CA, USA). A *p* value less than 0.05 was considered statistically significant.

## 3. Results

### 3.1. Ubiquinol Promotes RGC Survival in Glaucomatous DBA/2J Mice

**Figure 1:**
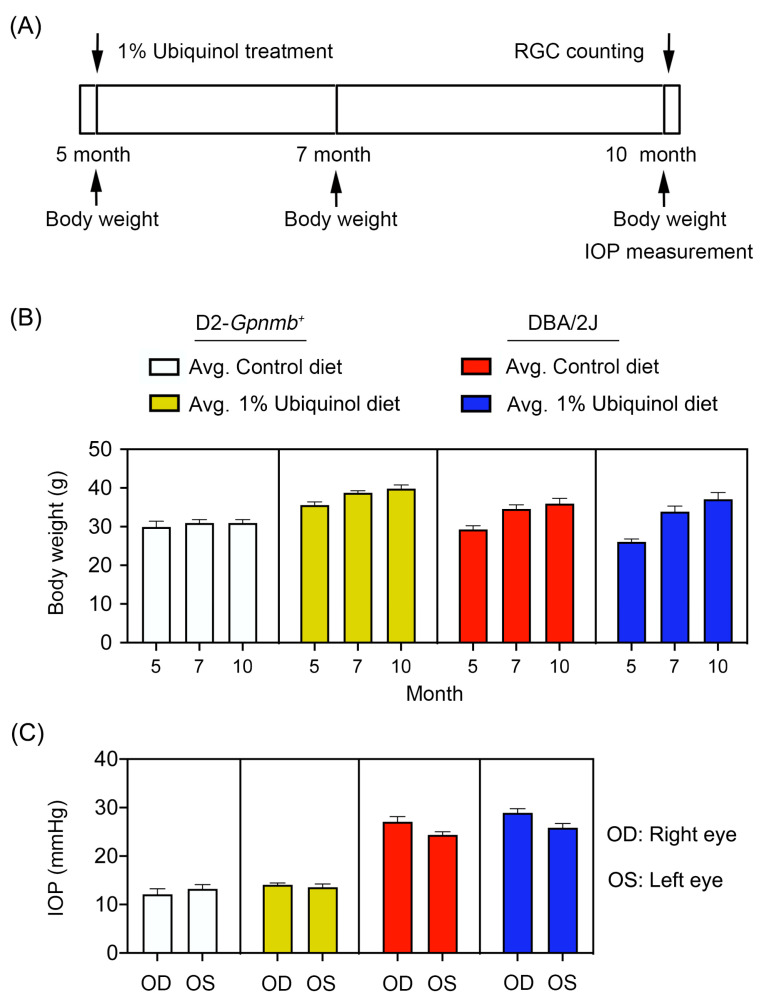
Ubiquinol supplementation and intraocular pressure (IOP) measurement in glaucomatous DBA/2J mice. (**A**) Diagram for control and ubiquinol diet (1%) supplementation and measurements for body weight, IOP and retinal ganglion cell (RGC) survival. Preglaucomatous DBA/2J and age-matched control D2-*Gpnmb^+^* mice at age 5 months were fed with either an unsupplemented control diet or ubiquinol diet for 5 months. The control and ubiquinol diet were given daily on chow. (**B**,**C**) Body weight (**B**) and IOP history (**C**) in control diet and ubiquinol diet-supplemented DBA/2J and age-matched D2-*Gpnmb^+^* mice. There was no significant change in the body weight and IOP among groups. Data are expressed as mean ± SEM (*n* = 20 for D2-*Gpnmb^+^* mice/group; *n* = 30 for DBA/2J mice/group).

Preglaucomatous DBA/2J mice that have not IOP elevation and age-matched nonglaucomatous control D2-*Gpnmb^+^* mice at age 5 months were fed with either unsupplemented control diet or ubiquinol (1%)-supplemented diet treatment daily for 5 months (Figure 1A). To determine the average of daily doses, we measured the total consumption of control diet and a diet of supplemented with ubiquinol per cage by weekly and averaged and recalculated the daily dose per mouse. We found that the average of daily dose of ubiquinol per mouse was 38 ± 4 mg in DBA/2J mice. We determined the body weight at age 5, 7, and 10 months, as well as the IOP at age 10 months (Figure 1). No significant change was observed in the body weight between control and ubiquinol diet-treated glaucomatous DBA/2J mice (Figure 1B). Our previous study demonstrated that the percentage of glaucomatous DBA/2J mice that have confirmed IOP elevation was 65.3% (64/98) at 10 months of age using an invasive method [16]. Using the tonometer, we found that the mean IOP was 26 ± 1.8 mm Hg in 10-month-old glaucomatous DBA/2J mice and that the percentage of glaucomatous DBA/2J mice that exceeded more than 25 mmHg was 53.3% (32/60) at 10 months of age. In addition, there were no significant changes in the IOPs between control diet and ubiquinol diet-supplemented glaucomatous DBA/2J mice (Figure 1C), indicating that the ubiquinol diet supplementation did not affect the change of IOPs in DBA/2J mice during glaucoma progression. The mean IOP was 13 ± 0.8 mm Hg in age-matched D2-*Gpnmb^+^* mice (Figure 1C).

**Figure 2:**
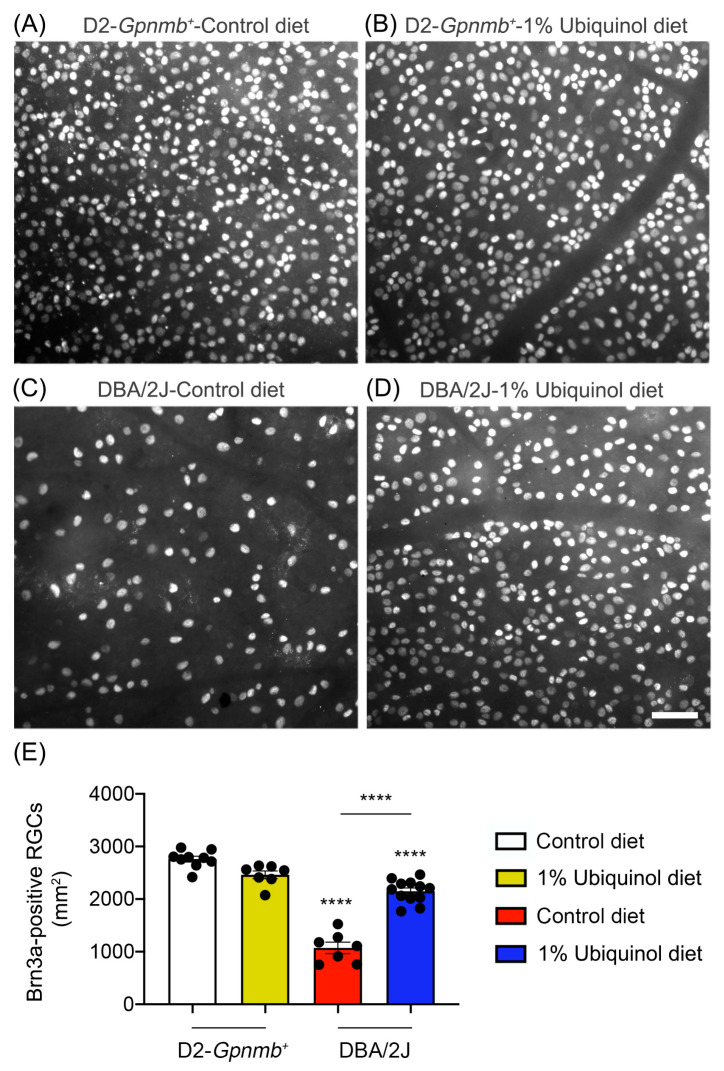
Effect of ubiquinol on RGC survival in glaucomatous DBA/2J mice. (**A**–**D**) Retinal whole-mount immunohistochemistry using Brn3a antibody. Representative images showed Brn3a-positive RGCs in the middle areas of the retinas from each group. (**E**) Quantitative analysis of RGC survival. Data are expressed as mean ± SEM (*n* = 7 retinas/group). **** *p* < 0.0001 (One-way ANOVA with the Bonferroni post hoc test) compared with 10 month-old D2-*Gpnmb^+^* mice supplemented with control diet. Scale bar 100 μm.

We determined whether ubiquinol diet supplementation promotes RGC survival in glaucomatous DBA/2J mice by Brn3a immunohistochemistry. Mean RGC density in the middle area per retina for each group was presented and summarized in Table 1. Nonglaucomatous control D2-*Gpnmb^+^* mice had an average of 2755 ± 53 RGCs in the middle (*n* = 7 to 9 retinas) (Figure 2A,E, and Table 1). In comparison with control D2-*Gpnmb^+^* retina supplemented with control diet, ubiquinol diet supplementation showed no significant difference in control D2-*Gpnmb^+^* retina (Figure 2B,E, and Table 1). However, control diet supplementation showed a significant RGC loss by an approximate 50% reduction in the retina of glaucomatous DBA/2J mice (Figure 2C,E, and Table 1). In contrast, ubiquinol diet supplementation significantly promoted RGC survival by an approximate 50% in the retina of glaucomatous DBA/2J mice compared with control diet-supplemented glaucomatous DBA/2J retina (Figure 2D,E, and Table 1).

### 3.2. Ubiquinol Blocks BAX Activation and Regulates Mitochondrial Biogenesis in the Retina of Glaucomatous DBA/2J Mice

**Figure 3:**
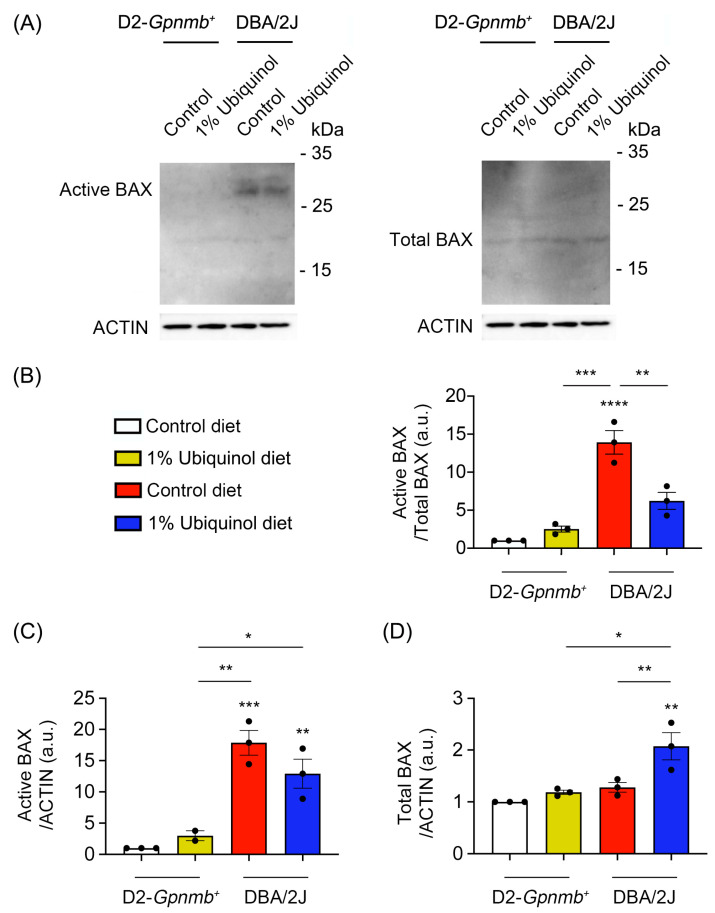
Effect of ubiquinol on BAX in the retina of glaucomatous DBA/2J mice. (**A**–**D**). Western blot analysis for active and total BAX protein expression in the retina. Data are expressed as mean ± SEM (*n* = 4 retinas/mice/group). * p < 0.05, ** *p* < 0.01, *** *p* < 0.001 and **** p < 0.0001 (One-way ANOVA with the Bonferroni post hoc test).

**Figure 4:**
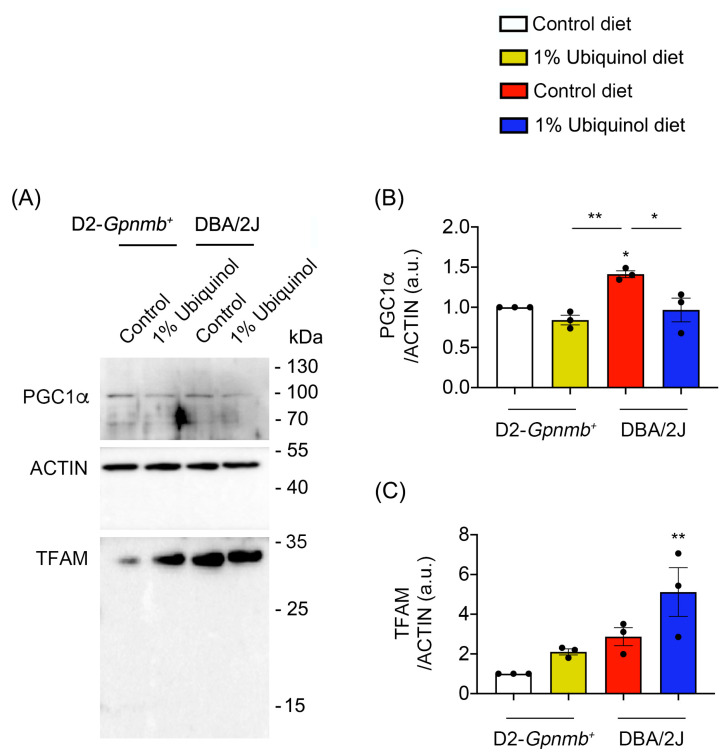
Effect of ubiquinol on PGC-1α and transcription factor A (TFAM) in the retina of glaucomatous DBA/2J mice. (**A**–**C**) Western blot analysis for PGC-1α and TFAM protein expression in the retina. Data are expressed as mean ± SEM (*n* = 4 retinas/mice/group). * p < 0.05 and ** *p* < 0.01 (One-way ANOVA with the Bonferroni post hoc test).

CoQ_10_ decreases total BAX protein expression in glaucomatous and ischemic retinas [7,8] and increases mitochondrial mass in ONH astrocytes against oxidative stress in vitro [9]. We investigated whether ubiquinol diet supplementation blocks BAX activation and regulates mitochondrial biogenesis in the retina of glaucomatous DBA/2J mice. At age 10 months, we performed Western blot analyses using antibodies raised against total and active BAX, PGC-1α and TFAM. In comparison with control diet-treated D2-*Gpnmb^+^* retina, control diet supplementation showed a significant increase of active BAX protein expression by 13.92 ± 1.55-fold in glaucomatous DBA/2J retina (Figure 3A–D). However, ubiquinol diet supplementation showed a significant decrease of active BAX protein expression by 6.22 ± 1.11-fold in glaucomatous DBA/2J retina compared with control diet-supplemented glaucomatous DBA/2J retina (Figure 3A–D). In comparison with control diet-treated D2-*Gpnmb^+^* retina, control diet supplementation showed a significant increase of PGC-1α protein expression by 1.41 ± 0.04-fold in glaucomatous DBA/2J retina (Figure 4A,B). However, ubiquinol diet supplementation showed a significant decrease of PGC-1α protein expression by 0.97 ± 0.15-fold in glaucomatous DBA/2J retina compared with control diet-supplemented glaucomatous DBA/2J retina (Figure 4A,B). Interestingly, ubiquinol diet supplementation also showed a significant increase of TFAM protein expression by 5.11 ± 1.22-fold in glaucomatous DBA/2J retina compared with control diet- or ubiquinol diet-supplemented D2-*Gpnmb^+^* retina (Figure 4A,C).

### 3.3. Ubiquinol Increases Expression Level of OXPHOS Complex II in the Retina of Glaucomatous DBA/2J Mice

**Figure 5:**
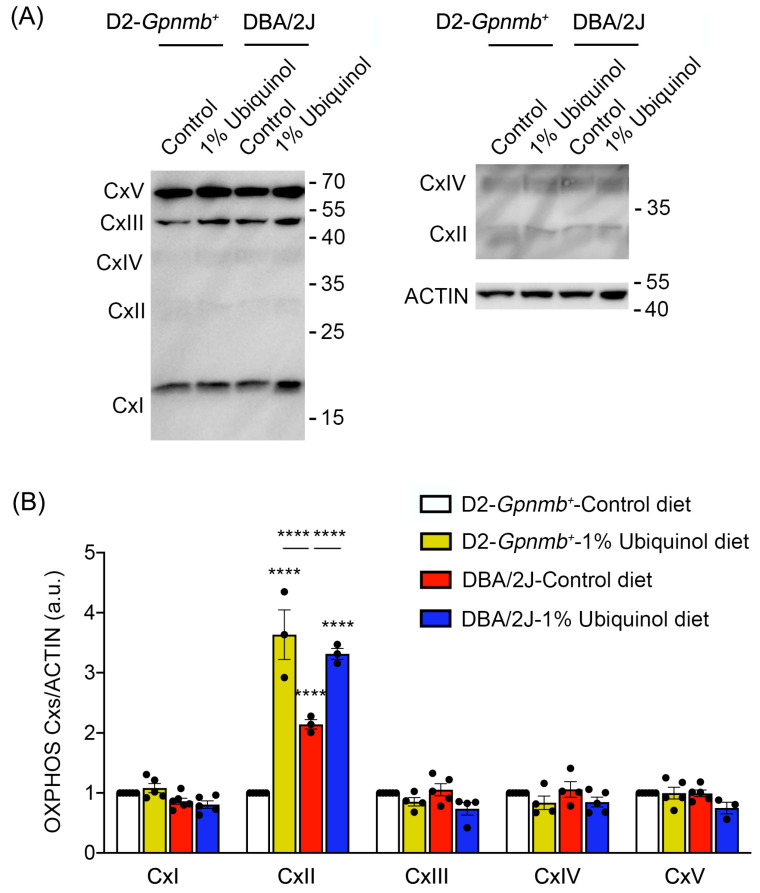
Effect of ubiquinol on oxidative phosphorylation (OXPHOS) complexes in the retina of glaucomatous DBA/2J mice. (**A**,**B**) Western blot analysis for OXPHOS complexes protein expression in the retina. Data are expressed as mean ± SEM (*n* = 4 retinas/mice/group). **** p < 0.0001 (two-way ANOVA with the Bonferroni post hoc test).

CoQ_10_ preserves not only OXPHOS complexes I and II in ONH astrocytes against oxidative stress in vitro [9] but also OXPHOS complex IV in glaucomatous retina [8]. We next investigated whether ubiquinol diet supplementation regulates OXPHOS complexes in the retina of glaucomatous DBA/2J mice. At age 10 months, we performed Western blot analysis using an OXPHOS complex. In comparison with control diet-treated D2-*Gpnmb^+^* retina, ubiquinol diet supplementation showed a significant increase of OXPHOS complex II protein expression by 3.63 ± 0.41-fold in D2-*Gpnmb^+^* retina (Figure 5A,B). In comparison with control diet-treated D2-*Gpnmb^+^* retina, control diet supplementation also showed a significant increase of OXPHOS complex II protein expression by 2.14 ± 0.07-fold in glaucomatous DBA/2J retina (Figure 5A,B). However, ubiquinol diet supplementation showed a significant increase of OXPHOS complex II protein expression by 3.31 ± 0.09-fold in glaucomatous DBA/2J retina compared with control diet-supplemented glaucomatous DBA/2J retina (Figure 5). In addition, there were no statistically significant differences in OXPHOS complexes I and III-V between control diet and ubiquinol diet-supplemented groups (Figure 5A,B).

### 3.4. Ubiquinol Protects RGCs against Oxidative Stress

**Figure 6:**
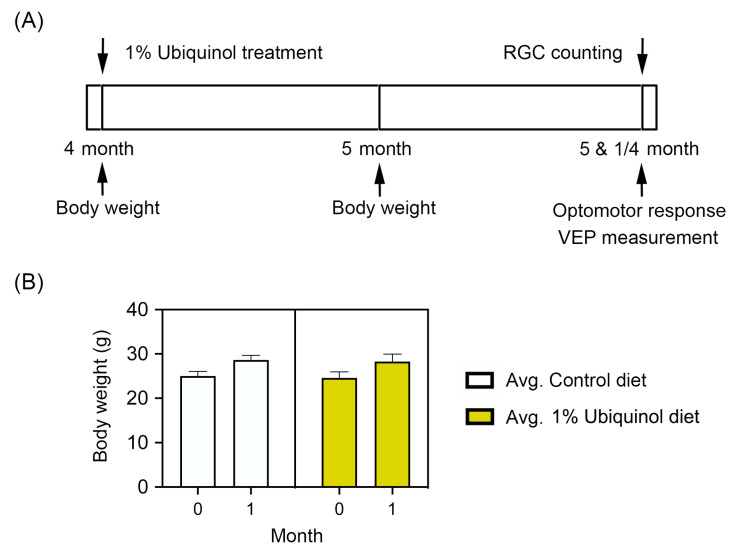
Ubiquinol supplementation in paraquat (PQ)-induced C57BL/6J mice. (**A**) Diagram for control and ubiquinol diet (1%) supplementation and measurements for body weight, optomotor response, visual evoked potential (VEP) and RGC survival. Four month old C57BL/6J mice were fed with either an unsupplemented control diet or the ubiquinol diet daily for 1 month. The control and ubiquinol diet were given daily on chow. (**B**) Body weight in control and ubiquinol diet-treated mice. Data are expressed as mean ± SEM (*n* = 10 for C57BL/6J mice/group).

**Figure 7:**
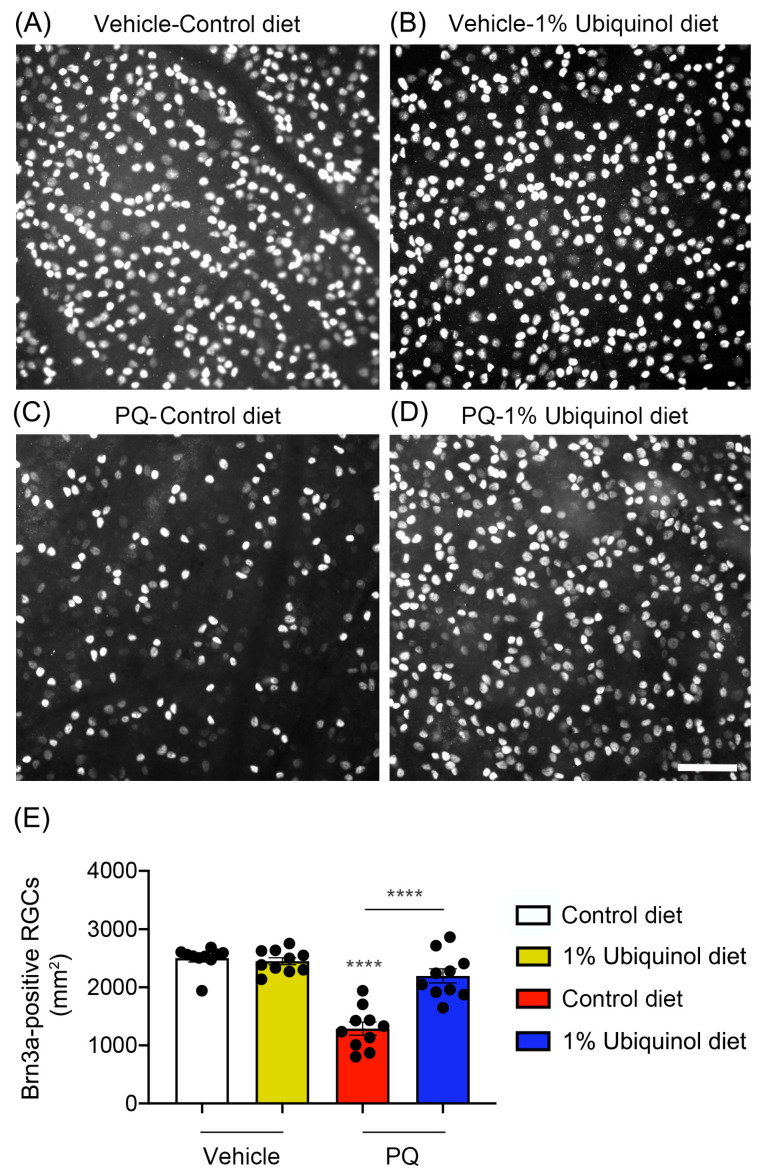
Effect of ubiquinol on RGC survival in PQ-induced C57BL/6J mice. (**A**–**D**) Retinal whole-mount immunohistochemistry using an antibody raised against Brn3a. Representative images showed Brn3a-positive RGCs in the middle areas of the retinas from each group. (**E**) Quantitative analysis of RGC survival. Data are expressed as mean ± SEM (*n* = 5 retinas/group). **** *p* < 0.0001 (One-way ANOVA with the Bonferroni post hoc test) compared with control C57BL/6J mice supplemented with control diet. Scale bar 100 μm.

C57BL/6J mice at age 4 months were fed either unsupplemented control diet or ubiquinol (1%) diet supplementation daily for 1 month before the induction of oxidative stress by PQ injection and then continued diet treatment for 1 week (Figure 6A). We found that the daily dose for ubiquinol per mouse was 33 ± 3 mg in C57BL/6J mice. By measuring the body weight at age 4 and 5 months, no significant change was observed in the body weight between control diet and ubiquinol diet-supplemented C57BL/6J mice (Figure 6B). We determined whether ubiquinol diet supplementation promotes RGC survival in PQ C57BL/6J mice by Brn3a immunohistochemistry. Mean RGC density in the middle area per retina for each group was presented and summarized in Table 2. Non-PQ control C57BL/6J mice had an average of 2514 ± 56 RGCs in the middle (*n* = 5 retinas/group) (Figure 7A,E, and Table 2). In comparison with non-PQ control retina supplemented with control diet, ubiquinol diet supplementation showed no significant difference in non-PQ control retina (Figure 7B,E, and Table 2). However, control diet supplementation showed a significant RGC loss by an approximate 49% reduction in PQ-injected retina (Figure 7C,E, and Table 2). In contrast, ubiquinol diet supplementation significantly promoted RGC survival by an approximate 40% in PQ-injected retina compared with control diet-supplemented PQ-injected retina (Figure 7D,E, and Table 2).

### 3.5. Ubiquinol Ameliorates Visual Dysfunction against Oxidative Stress

**Figure 8:**
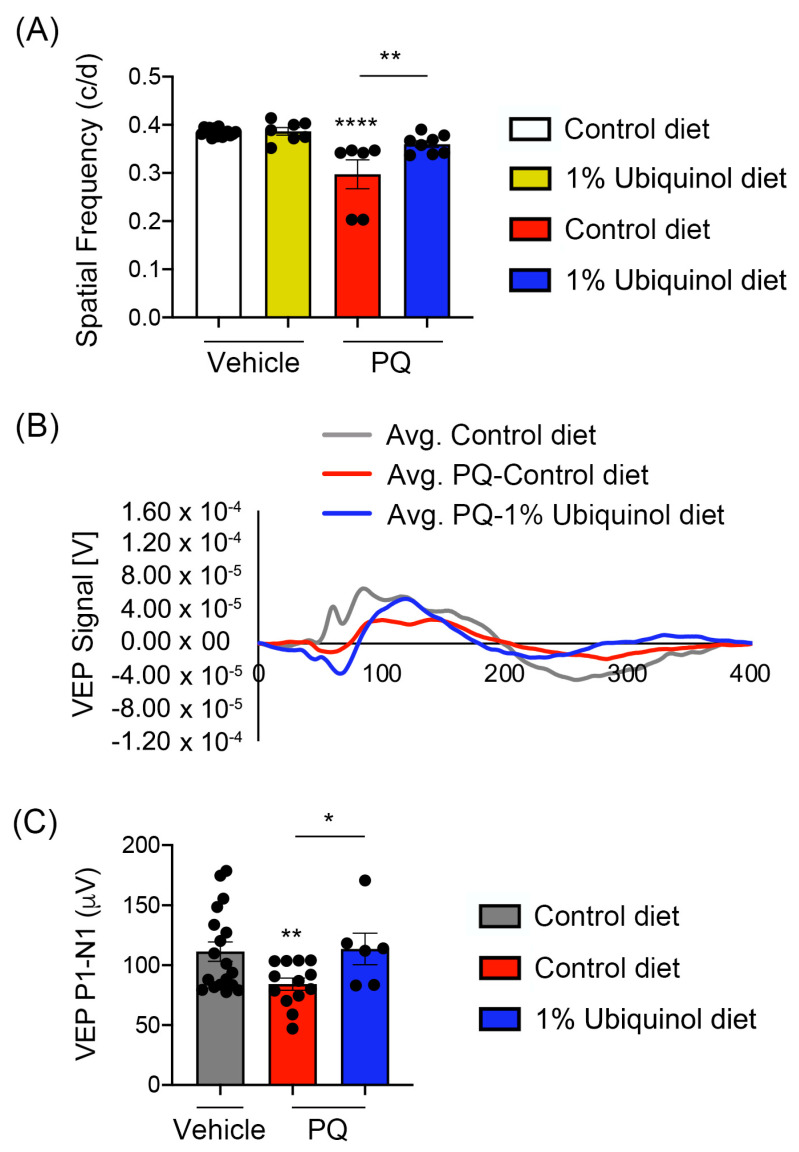
Effect of ubiquinol on visual function in PQ-induced C57BL/6J mice. (**A**) Visual function test by optomotor response analyses. Data are expressed as mean ± SEM (*n* = 6 to 9 mice/group). ** *p* < 0.01 and **** *p* < 0.0001 (One-way ANOVA with the Bonferroni post hoc test). (**B**) Total recordings of VEP responses. (**C**) Visual function test by VEP analyses. Note that there was a significant preservation of VEP in ubiquinol-supplemented C57BL/6J mice against oxidative stress. Data are expressed as mean ± SEM (*n* = 12 to 22 mice/group). * *p* < 0.05 and ** *p* < 0.01 (One-way ANOVA with the Bonferroni post hoc test).

We determined whether ubiquinol diet supplementation ameliorates visual dysfunction in oxidative stress-induced C57BL/6J mice by visual function tests. We measured (1) the maximum spatial frequency in a virtual-reality optomotor system and (2) VEP-mediated central visual function. In comparison with non-PQ control C57BL/6J mice supplemented with control diet or ubiquinol diet, we found a significant reduction of optomotor responses in PQ-induced C57BL/6J mice supplemented with control diet by decreasing spatial frequency (Figure 8A). In contrast, ubiquinol diet supplementation significantly preserved optomotor responses compared with PQ-injected C57BL/6J mice supplemented with control diet (Figure 8A). Consistent with this finding, we also found a significant reduction of VEP P1-N1 potentials in PQ-injected C57BL/6J mice supplemented with control diet (Figure 8B,C). In addition, ubiquinol diet supplementation significantly preserved VEP P1-N1 potentials compared with PQ-injected C57BL/6J mice supplemented with control diet (Figure 8B,C).

## 4. Discussion

CoQ_10_ is a neurotherapeutic agent and potent antioxidant against mitochondrial dysfunction and oxidative stress in experimental glaucoma [8,10,13,22,23,24,25,26]. In the present study, we demonstrated that a diet supplementation with ubiquinol (1%) significantly enhanced RGC survival in a chronic mouse model of glaucoma, DBA/2J mice. In parallel, we found ubiquinol contributed to RGC survival via modulating mitochondrial biogenesis, OXPHOS complex and BAX activation in glaucomatous DBA/2J mice. Additionally, ubiquinol ameliorated RGC death and visual dysfunction in mice against oxidative stress. Here, we suggest for the first time that ubiquinol would be a therapeutic antioxidant for preventing mitochondrial dysfunction and oxidative stress in glaucomatous RGC degeneration.

It has demonstrated that CoQ_10_ level in the human retina declined by approximately 40% with aging [27]. Interestingly, our recent study showed that CoQ_10_ protected RGCs against elevated IOP-induced retinal degeneration in aged glaucomatous DBA/2J mice [8], suggesting that the reduction in CoQ_10_ level with age may result in increased susceptibility to RGCs during glaucoma progression due to aging being a significant risk factor for the prevalence of glaucoma. Importantly, it has been shown that biosynthesis and levels of ubiquinol are decreased in aging tissues [28]. Emerging evidence from our group demonstrated that ubiquinol significantly promoted RGC survival and blocked apoptotic pathway in acute IOP elevation-induced ischemic retinal degeneration [14], raising the possibility that ubiquinol may promote RGC survival in aged glaucomatous neurodegeneration. Indeed, the current results strongly demonstrated that ubiquinol is protective to RGCs in aged glaucomatous DBA/2J mice. BAX is critical to many apoptotic pathways [29,30] and it is interacting with the element composing the mitochondrial permeability transition pore that allows proteins to escape from the mitochondria into the cytosol to initiate apoptosis [31,32,33]. Our previous study has demonstrated that CoQ_10_ inhibited the mitochondria-related apoptotic pathway in ischemic and glaucomatous retinas by modulating BAX expression and BAD phosphorylation [7,8]. Hence, our current finding of reduced active BAX expression by ubiquinol supplementation suggests that ubiquinol protects RGC mitochondria by regulating the BAX/BAD/BCL-xL pathway in glaucomatous neurodegeneration.

PGC-1α activates oxidative metabolism and mitochondrial biogenesis [34,35], as well as regulates the transcription-related proteins such as TFAM, which has a critical role in mitochondrial DNA (mtDNA) maintenance and mitochondrial gene expression [34,36,37]. It has been implicated that alterations of mtDNA induced by oxidative stress are associated with glaucoma pathogenesis [38,39,40,41,42,43,44,45]. TFAM controls mtDNA copy numbers in mammals and its levels are correlated with the levels of mtDNA [46,47]. Recent study suggests that TFAM is rapidly upregulated in the early neurodegenerative events of ischemic retina or neonatal hypoxic-ischemic brain, suggesting that TFAM may be associated with the endogenous repair mechanism of damaged retinal or brain neurons [48,49]. Our previous study has demonstrated that CoQ_10_ promoted PGC-1α protein expression in ONH astrocytes against oxidative stress and preserved TFAM protein expression in glaucomatous retina [8]. Of interest, our current findings demonstrated that ubiquinol supplementation dramatically enhanced TFAM protein expression in glaucomatous retina, while the expression level of PGC-1α protein was preserved in glaucomatous retina. In mice, human TFAM overexpression enhances the amount of mtDNA and prevents the cardiac dysfunctions induced by myocardial infarction [50]. Because activation of PGC-1α correlates with increased cellular energy demand [51], it is likely that ubiquinol protects RGCs via maintaining or increasing mtDNA in glaucomatous DBA/2J mice, in turn, leads to the preservation of PGC-1α expression. A previous study demonstrated that PGC-1α expression did not correlate with the expression level of TFAM [52] even though PGC-1α controls transcriptional expression of TFAM. Therefore, differential expression of PGC-1α and TFAM in glaucomatous retina suggests that ubiquinol may be associated with other transcriptional cofactors. Further studies will determine whether ubiquinol promotes TFAM-mediated mitochondrial biogenesis in RGCs via increasing mtDNA copy number against glaucomatous insults.

In the current study, we found that ubiquinol supplementation significantly increased the expression level of OXPHOS complex II protein in control non-glaucomatous retina. Of note, ubiquinol supplementation significantly enhanced OXPHOS complex II expression in glaucomatous retina compared to control diet supplementation. Recent evidence indicates that mitochondrial complex II, which is a source of the spare or reserve respiratory capacity (RRC), promotes cell survival [53]. The enhanced RRC is linked to cell death-resistance and survival [54] whereas the reduced RRC is associated with neuronal cell death [55]. Because the evidence suggests that RRC and complex II promote cell survival after energy deprivation [53], we propose the possibility that enhanced OXPHOS complex II by ubiquinol supplementation may be associated with RGC survival against elevated IOP-mediated energy deprivation or crisis. Indeed, our previous study demonstrated that elevated IOP-mediated oxidative stress triggered extensive mitochondrial fragmentation and cristae depletion in glaucomatous RGC soma and axons [15,16]. Additionally, recent studies indicated that elevated IOP induced the reduction of energy capacity and the limitation of responsiveness to increased energy demand in glaucomatous RGC axons [56,57].

Oxidative stress is considered a major causal factor for mitochondrial dysfunction and glaucomatous neurodegeneration [8,15,45,58,59,60,61,62,63]. Since our previous studies demonstrated that CoQ_10_ protected RGCs in vivo or ONH astrocytes in vitro against oxidative stress-induced mitochondrial dysfunction [7,8,9], our recent evidence indicated that ubiquinol protects RGCs by inhibiting BAX-mediated apoptotic pathway in ischemic retina. Thus, we propose that ubiquinol is critical to ameliorate oxidative stress-mediated RGC death. In the current study, we further demonstrated the effect of ubiquinol supplementation on RGC survival and vision preservation against PQ-induced oxidative stress. To determine the direct protective effect of ubiquinol supplementation on oxidative stress-induced RGC death and visual dysfunction, we administrated PQ, an oxidative stress inducer, which induces mitochondrial oxidative damage by increasing superoxide radical production [64,65]. It has been reported that PQ administration triggered retinal degeneration and RGC death [66,67], as well as loss of virtual optomotor response [68,69]. However, the effect of ubiquinol on RGC survival and visual function against PQ-induced oxidative stress remained unknown. In the current study, we demonstrated that ubiquinol supplementation protects RGCs in vivo and preserved visual function against PQ-induce oxidative stress. Of interest, CoQ_10_ is neuroprotective in PQ-induced experimental models of Parkinson’s disease [70,71,72] and differentiated human neuroblastoma (SH-SY5Y) cells in vitro [72]. Given our findings and those of others, it is conceivable that ubiquinol supplementation promotes RGC survival and has therapeutic potential for ameliorating elevated IOP-induced and/or oxidative stress-induced RGC death and visual dysfunction.

## 5. Conclusions

Our findings demonstrate that ubiquinol protects RGCs by modulating the BAX-mediated apoptotic pathway and by increasing TFAM expression and OXPHOS complex II activity in glaucomatous neurodegeneration. In addition, we found that ubiquinol enhances RGC survival and preserves visual function against oxidative stress. Thus, we propose that ubiquinol has a therapeutic potential for treating oxidative stress-associated glaucomatous neurodegeneration.

## Figures and Tables

**Table 1:** Effect of ubiquinol on Brn3a-positive RGCs in the middle area of glaucomatous DBA/2J retinas.

Experimental Group	Brn3a-Positive RGCs
Nonglaucomatous D2-*Gpnmb^+^* mice/Control diet	2755 ± 53
Nonglaucomatous D2-*Gpnmb^+^* mice/Ubiquinol diet	2461 ± 72
Glaucomatous DBA/2J mice/Control diet	1072 ± 78 ****
Glaucomatous DBA/2J mice/Ubiquinol diet	2150 ± 61 ****

Data are expressed as the mean ± SEM (*n* = 7 to 9 retinal flatmounts/experimental group). **** *p* < 0.0001 (One-way ANOVA with the Bonferroni post hoc test) compared with 10 month-old D2-*Gpnmb^+^* mice supplemented with control diet or 10 month-old glaucomatous DBA/2J mice supplemented with control diet.

**Table 2:** Effect of ubiquinol on Brn3a-positive RGCs in the middle area of oxidative stress-induced C57BL/6J retinas.

Experimental Group	Brn3a-Positive RGCs
Control C57BL/6J/Control diet	2514 ± 56
Control C57BL/6J/Ubiquinol diet	2464 ± 50
PQ-treated C57BL/6J/Control diet	1290 ± 106 ****
PQ-treated C57BL/6J/Ubiquinol diet	2164 ± 106 ****

Data are expressed as the mean ± SEM (*n* = 5 retinal flatmounts/experimental group). **** *p* < 0.0001 (One-way ANOVA with the Bonferroni post hoc test) compared with control C57BL/6J mice supplemented with control diet or PQ-induced C57BL/6J mice supplemented with control diet.
